# Supplementary material for: Selective Isolation of Liver-Derived Extracellular Vesicles Redefines Performance of miRNA Biomarkers for Non-Alcoholic Fatty Liver Disease
Source: Biomedicines. 2022 Jan 17;10(1):195. doi: 10.3390/biomedicines10010195 (PMC8773667; doi:10.3390/biomedicines10010195)
Supplement: Supplementary file 1 [file biomedicines-10-00195-s001.zip › biomedicines-1442767-supplementary.pdf]

SUPPLEMENTARY DATA

SELECTIVE ISOLATION OF LIVER DERIVED EXTRACELLULAR VESICLES ENHANCES THE PERFORMANCE OF MIRNA BIOMARKERS FOR NON-ALCOHOLIC FATTY LIVER DISEASE.

Lauren A Newman[1], Zivile Useckaite[1], Jillian Johnson[2], Michael J Sorich[1], Ashley M Hopkins[1] and Andrew Rowland[1*]

[1] College of Medicine and Public Health, Flinders University, Adelaide, South Australia
[2] Early Clinical Development, Pfizer Global Research and Development Groton CT

[*] Corresponding author: Andrew Rowland, College of Medicine and Public Health, Flinders University, Adelaide, South Australia, 5042. Phone: +61 8 8204 7546, email: andrew.rowland@flinders.edu.au

(A1) Control – 30,000 x

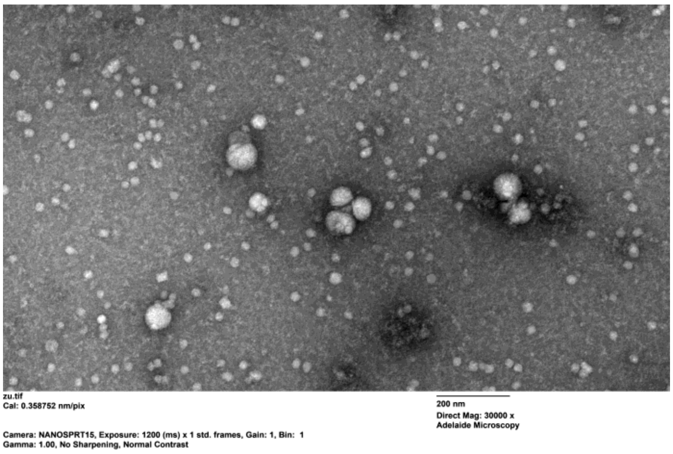

(A2) Control – 68,000 x

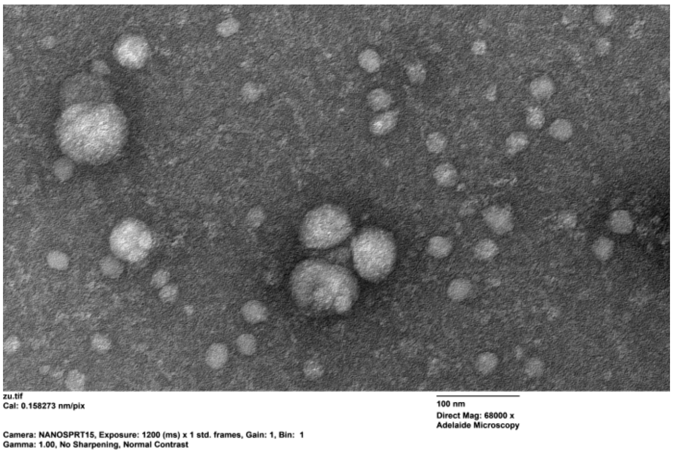

(B1) NASH – 30,000 x

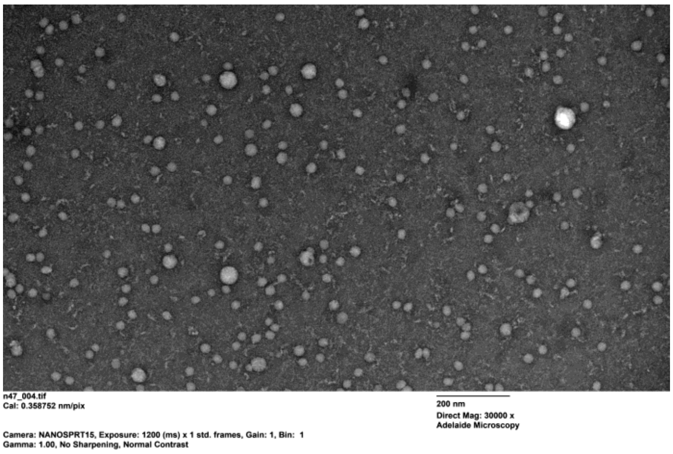

(B2) NASH – 68,000 x

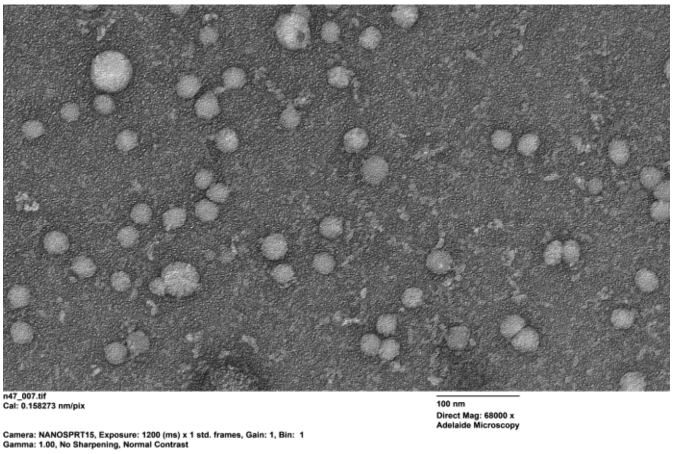

Figure S1. Unedited TEM images at 30,000 x magnification (A1, B1) and at 68,000 x magnification (A2, B2).

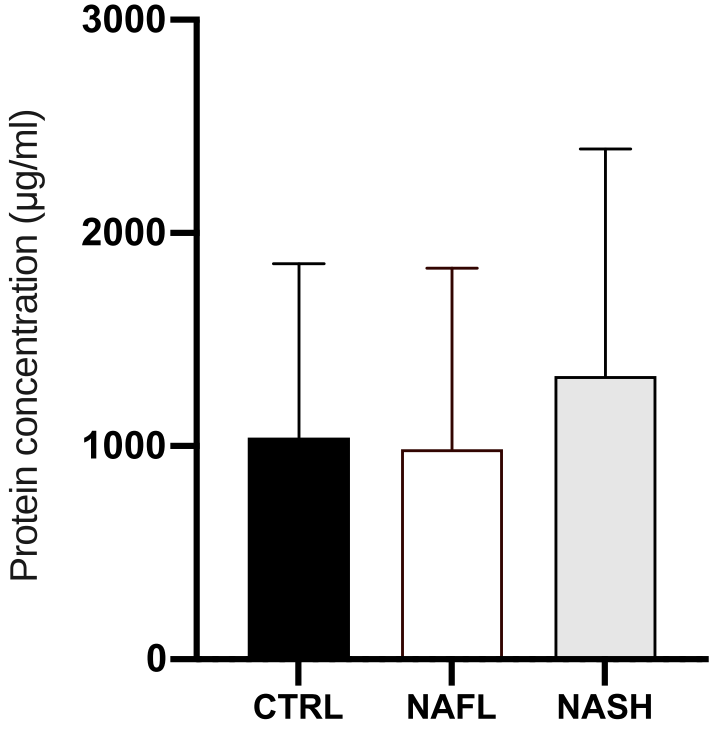

Figure S2. Total protein concentration in global EVs isolated from control, NAFL and NASH subjects, determined using Pierce Micro BCA Protein Assay. Data shown as mean ± SD.

Characterisation of EVs isolated by anti-ASGR1 immunoprecipitation

Global EVs were isolated from healthy subject plasma (n=5) and liver-specific EVs captured via anti-ASGR1 immunoprecipitation as described in Materials and Methods. Non-captured EVs were recovered following the 24hr incubation and separation of bead-bound EVs. To allow for NTA, captured vesicles were eluted from the beads by incubating with 1 volume of 0.2µm-filtered 0.1M glycine-HCl at pH 3 for 10 minutes. The beads were separated using the magnet and the supernatant neutralised with 0.1 volume of filtered 1M Tris-HCl. NTA, peptide digestion and LCMS was performed as described in Materials and Methods with some differences to detect ASGR1. Specifically, the analyte was eluted from the column by gradient separation. Starting conditions were 90% mobile phase A (0.1% formic acid in water) and 10% mobile phase B (0.1% formic acid in acetonitrile). Mobile phase B increased to 60% over 8 minutes, then returned to 10% over 1.4 minutes and re-equilibrated for 0.6 minutes for a total run time of 10 minutes.

Concentration of particles in global, captured and non-captured portions was determined by NTA and revealed that ASGR1+ EVs represented between 7.82 and 13.70 % of circulating EVs (Table S1). Total recovery (i.e. Captured + non-captured / global) was 82% on average. Losses may be attributed to washing steps following collection of the non-captured portion.

ASGR1 abundance was measured by LCMS in global EV, captured and non-captured samples. Repeated measures one-way ANOVA revealed that ASGR1 abundance did not differ significantly between matched global and IP isolates, whereas ASGR1 was beneath the limit of detection for non-captured fractions (Fig. S3). In all, these data show that our anti-ASGR1 immunoprecipitation procedure successfully recovers circulating EVs of liver origin.

Table S1 Particle concentration in global EV isolates, ASGR1 immunocapture and non-captured samples determined by nanoparticle tracking analysis, with percentage of ASGR1+ EV and total EVs recovered (captured + non-captured / global).

**Sample**	**Concentration (Particles/mL)**			**Recovery (%)**	
	Global	Captured	Non-captured	ASGR1+	Total
1	2.95E+10	3.91E+09	1.52E+10	13.25	64.78
2	1.11E+10	1.42E+09	8.33E+09	12.79	87.84
3	3.32E+10	4.55E+09	2.52E+10	13.70	89.61
4	2.11E+10	1.65E+09	1.56E+10	7.82	81.75
5	3.48E+10	2.95E+09	2.71E+10	8.48	86.35

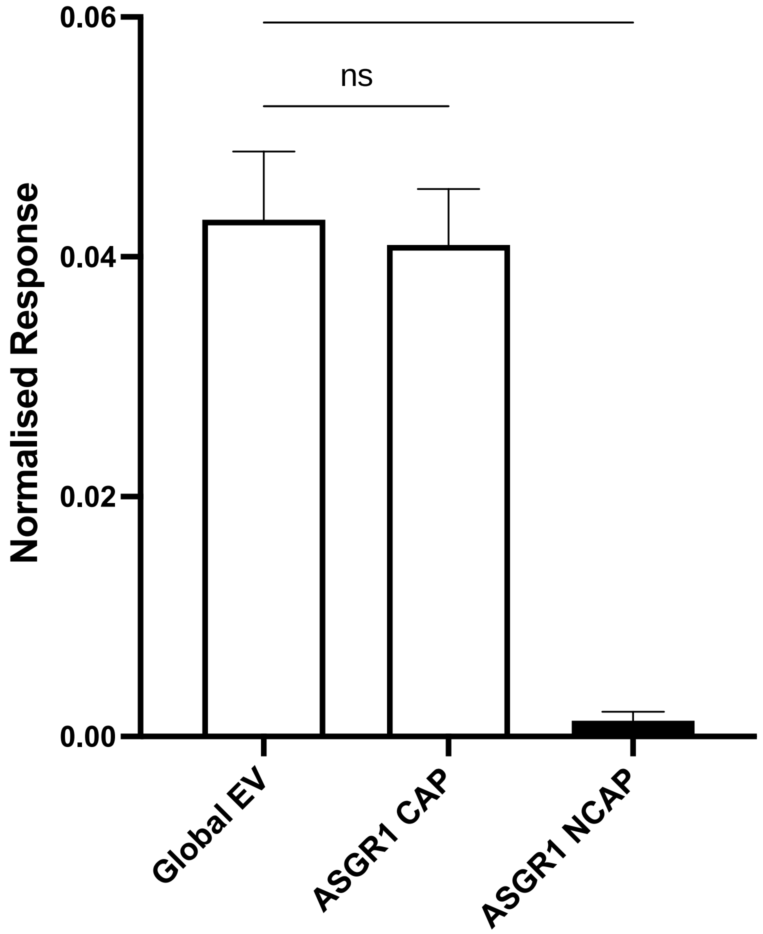

Figure S3. ASGR1 abundance (response normalised to stable isotope labelled peptide signal) in global EVs and anti-ASGR1 immunoprecipitation captured (CAP) and non-captured (NCAP) portions. Data shown as mean ± SD. Repeated measures one-way ANOVA with Dunnett's test for multiple comparisons: **** $p < 0.001$; ns, not significant.

Extended Receiver Operator Characteristic Curve Analysis

Receiver operator characteristic (ROC) analysis was undertaken to establish the performance of miRNA marker with respect to distinguishing individual paired groups (i.e. control vs NAFL, control vs NASH and NAFL vs NASH). ROC analyses were performed for total cell-free RNA and vesicular RNA from global and liver-specific EV fractions (Table S2; Fig. S4).

Consistent with prior reports[13], ROC analysis demonstrated strong performance of total plasma derived miRNAs with respect to distinguishing individual disease pairings. With the exception of miR-122 for NASH versus control (AUC=0.678, p=0.213) and miR-192 for NASH vs NAFL (AUC=0.729, p=0.156), differences in the abundance of miR-122, miR-192 and miR-128-3p were statistically significant between disease groups, with ROC values ≥ 0.833. In contrast, ROC analysis demonstrated comparatively poor performance of global EVs with respect to distinguishing individual disease pairings, with only miR-192 abundance in NASH versus control groups reaching statistical significance (ROC=0.989, p=0.001). Similarly, differences in liver-specific EV-derived miRNAs between individual pairings failed to reach statistical significance for several pairings (Table S2).

Table S2 Area Under the Receiver Operator Characteristic Curve (AUC) for miRNA biomarkers isolated from total cell-free RNA, global EVs and ASGR1+ EVs in distinguishing NAFL, NASH and control groups. * denotes significant p values (≤0.05).

	**miR-122**					
	NAFL–CTRL		NASH–CTRL		NASH–NAFL	
	AUC	p	AUC	p	AUC	p
**Total RNA**	0.833	0.010*	0.678	0.213	0.938	0.007*
**Global EV**	0.533	0.796	0.544	0.756	0.500	0.999
**ASGR1+ EV**	0.741	0.065	0.881	0.008*	0.688	0.245
	**miR-192**					
	NAFL–CTRL		NASH–CTRL		NASH–NAFL	
	AUC	p	AUC	p	AUC	p
**Total RNA**	0.858	0.006*	0.844	0.016*	0.729	0.156
**Global EV**	0.650	0.245	0.511	0.938	0.625	0.439
**ASGR1+ EV**	0.850	0.048*	0.925	0.006*	0.625	0.522
	**miR-128-3p**					
	NAFL–CTRL		NASH–CTRL		NASH–NAFL	
	AUC	p	AUC	p	AUC	p
**Total RNA**	0.917	0.001*	0.933	0.002*	0.875	0.020*
**Global EV**	0.800	0.020*	0.989	0.001*	0.688	0.245
**ASGR1+ EV**	0.677	0.257	0.846	0.018*	0.667	0.361

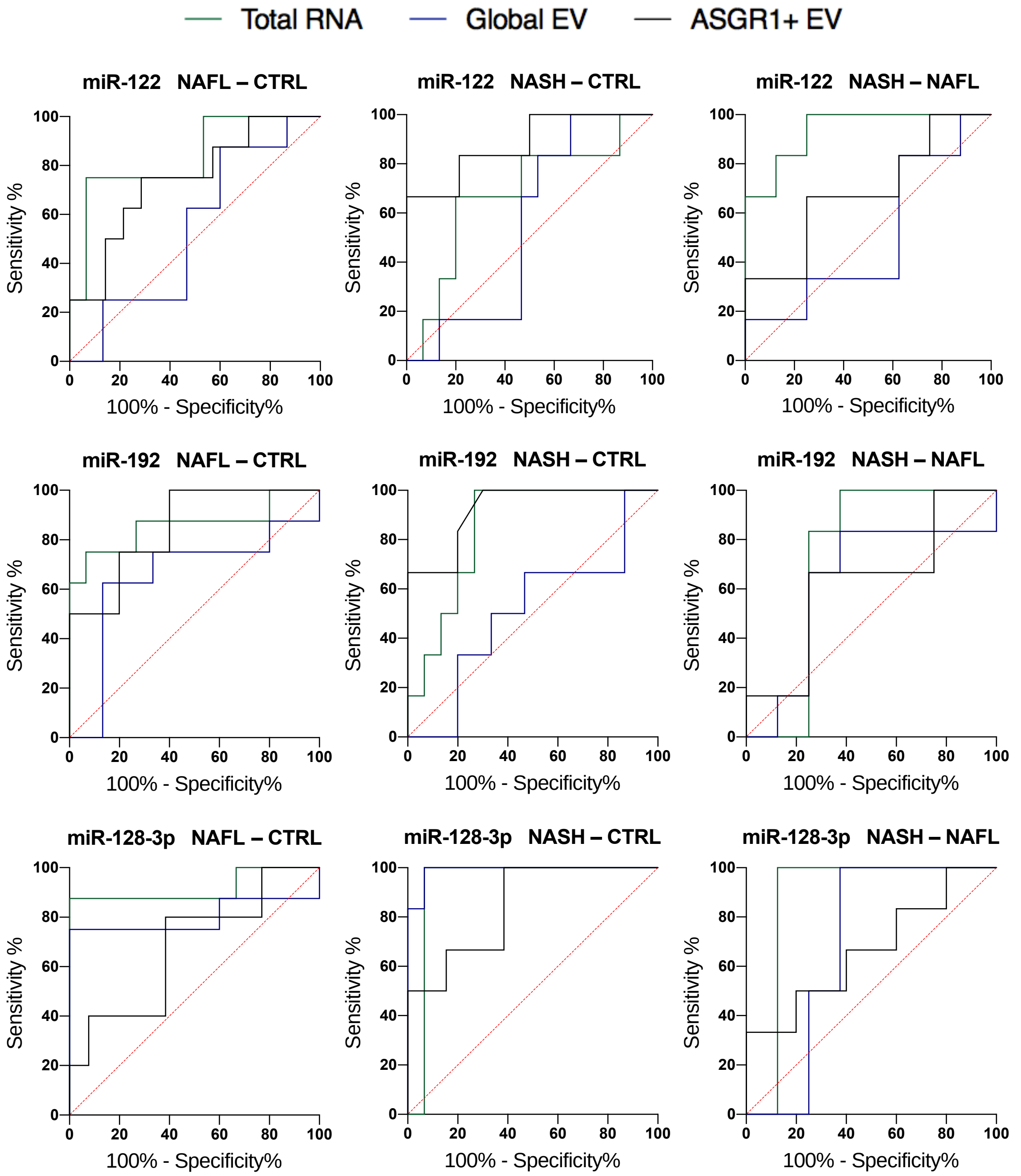

Figure S4. Receiver Operator Characteristic (ROC) curve analysis of miR-122, miR-192 and miR-128-3p in total cell-free, global EV and ASGR1+ EV RNA for distinguishing subjects in pairwise groupings (NAFL-CTRL, NASH-CTRL and NAFL-NASH).
